# Late Endosomal/Lysosomal Cholesterol Accumulation Is a Host Cell-Protective Mechanism Inhibiting Endosomal Escape of Influenza A Virus

**DOI:** 10.1128/mBio.01345-18

**Published:** 2018-07-24

**Authors:** Alexander Kühnl, Agnes Musiol, Nicole Heitzig, Danielle E. Johnson, Christina Ehrhardt, Thomas Grewal, Volker Gerke, Stephan Ludwig, Ursula Rescher

**Affiliations:** a Institute of Medical Biochemistry, Center for Molecular Biology of Inflammation, University of Muenster, Muenster, Germany; b Institute of Virology, Center for Molecular Biology of Inflammation, University of Muenster, Muenster, Germany; c Interdisciplinary Centre for Clinical Research, University of Muenster, Muenster, Germany; d Cluster of Excellence “Cells in Motion,” University of Muenster, Muenster, Germany; e Program in Cell Biology, Hospital for Sick Children, Toronto, Ontario, Canada; f Faculty of Pharmacy A15, University of Sydney, Sydney, NSW, Australia; University of Geneva; Cornell University

**Keywords:** IFITM3, influenza A virus, annexin A6, interferon, late endosomal cholesterol content

## Abstract

To transfer the viral genome into the host cell cytoplasm, internalized influenza A virus (IAV) particles depend on the fusion of the IAV envelope with host endosomal membranes. The antiviral host interferon (IFN) response includes the upregulation of interferon-induced transmembrane protein 3 (IFITM3), which inhibits the release of the viral content into the cytosol. Although IFITM3 induction occurs concomitantly with late endosomal/lysosomal (LE/L) cholesterol accumulation, the functional significance of this process is not well understood. Here we report that LE/L cholesterol accumulation itself plays a pivotal role in the early antiviral defense. We demonstrate that inducing LE/L cholesterol accumulation is antiviral in non-IFN-primed cells, restricting incoming IAV particles and impairing mixing of IAV/endosomal membrane lipids. Our results establish a protective function of LE/L cholesterol accumulation and suggest endosomal cholesterol balance as a possible antiviral target.

## IMPORTANCE

With annual epidemics occurring in all parts of the world and the risk of global outbreaks, influenza A virus (IAV) infections remain a major threat to public health. Infected host cells detect viral components and mount an interferon (IFN)-mediated response to restrict virus propagation and spread of infection. Identification of cellular factors and underlying mechanisms that establish such an antiviral state can provide novel strategies for the development of antiviral drugs. The contribution of LE/L cholesterol levels, especially in the context of the IFN-induced antiviral response, has remained controversial so far. Here, we report that accumulation of cholesterol in the LE/L compartment contributes to the IFN-induced host cell defense against incoming IAV. Our results establish cholesterol accumulation in LE/L *per se* as a novel antiviral barrier and suggest the endosomal cholesterol balance as a putative druggable host cell factor in IAV infection.

## INTRODUCTION

Influenza A virus (IAV) is responsible for annual epidemics that cause major challenges, in terms of both morbidity and mortality, and IAV pandemics claimed millions of deaths worldwide in the past (1). Thus, IAV is a major public health threat. Identifying host cell factors and components that are exploited by the virus to promote replication might offer targets to develop novel strategies of treatment. Comprehensive knowledge about the underlying mechanisms of pathogen-host interaction and the induction of the antiviral host innate immune response is crucial.

Upon initial cell attachment via binding of the IAV glycoprotein hemagglutinin (HA) to sialic acid residues on host cell surface proteins, viral particles utilize the cellular endocytic machinery to enter the target cell. Viruses are subsequently trafficked through early endosomes (EE) to late endosomes/lysosomes (LE/L), where endosomal escape is thought to occur (2–4). A prerequisite for the efficient release of the viral genome into the host cell cytoplasm is the fusion of the viral envelope with endosomal membranes, triggered by acidic conditions within the LE/L. The lower pH leads to a conformational change in HA, and subsequent hydrophobic interactions of HA with the endosomal membrane cause the formation of a fusion pore, allowing cytosolic entry and transport of the viral ribonucleoproteins to the nucleus (3, 4).

Detection of viral components in infected cells via the host innate immune system elicits rapid induction and secretion of the antiviral interferon (IFN) cytokine family, leading to subsequent upregulation of a plethora of genes that help restrict IAV infection and spread. IFN-induced elevation of levels of the antiviral protein IFITM3 (5) has been demonstrated to interfere with the fusion of the viral envelope with the LE/L membrane (5–11). Interestingly, cells ectopically expressing IFITM3 also show aberrant late endosomal accumulation of cholesterol, a lipid known to control membrane sorting and dynamics in this compartment (6, 10). It has thus been suggested that LE/L cholesterol accumulation links the antiviral IFITM3 activity to LE/L membrane properties (10). However, the relevance of altered endosomal cholesterol levels in IFITM3-mediated viral restriction is heavily disputed (6,9–11), and the issue of whether treatment with interferon beta (IFN-β) induces changes in subcellular cholesterol pools has not been addressed so far (12).

Because our previous results indicate a strong impact of balanced endosomal cholesterol on the release and infectivity of IAV progeny (13), we therefore systematically evaluated the impact of enhanced LE/L cholesterol contents in the context of the host cell antiviral response. We report that LE/L cholesterol accumulation already interferes with IAV infection at the early step of endosomal escape, thus contributing to the IFN-induced host cell defense against incoming IAV, and that the protective function is promoted via IFITM3. To address the issue of whether blocked LE/L cholesterol egress acts as a cellular restriction factor for IAV replication independently of the IFN/IFITM3 axis, we induced LE/L cholesterol accumulation either through pharmacological inhibition of the LE/L cholesterol transporter NPC1, the protein affected in Niemann-Pick disease, or via overexpression of the LE/L cholesterol balancing protein annexin A6 (AnxA6), which results in a phenotype reminiscent of NPC1 deficiency (reviewed in reference 14). We show that this IFN-independent LE/L cholesterol accumulation did not affect IAV endosomal trafficking but did impair IAV cytosolic entry, most likely at the step of IAV/endosome membrane hemifusion, i.e., when lipid mixing prior to the release of the actual viral content occurs. Thus, our data support a model of LE/L cholesterol accumulation as a novel antiviral barrier and as a putative druggable host cell factor in IAV infection.

## RESULTS

### IFN induces endosomal/lysosomal cholesterol accumulation.

**FIG 1:**
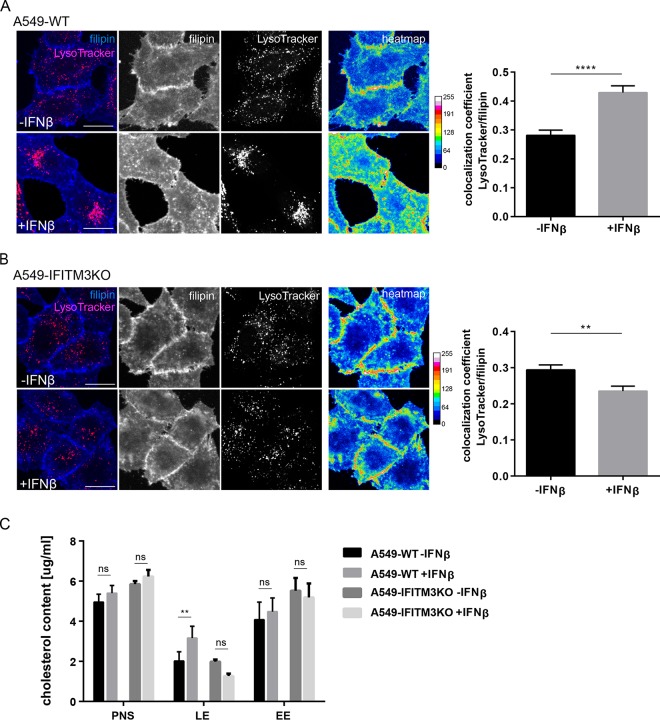
IFN-β induces cholesterol accumulation in acidic endosomes in A549 cells. (A) A549-WT cells or (B) A549-IFITM3KO cells were left untreated or exposed to IFN-β (10 ng/ml, 16 h) and loaded with LysoTracker Red DND-99 to label acidic endosomes. Unesterified cellular cholesterol was stained using filipin (blue). Filipin pixel intensities were pseudocolored according to the LUT and are shown as a heat map, with the gradient of colors ranging from blue (lowest intensity) to white (highest intensity). Bar, 20 µm. Colocalization coefficients of LysoTracker with filipin were quantitated from z-stacks. Mean values ± SEM were calculated from 35 individual cells per condition from at least two independent experiments. **, *P* ≤ 0.01; ****, *P* ≤ 0.0001 (unpaired Student’s *t* test). (C) A549-WT and A549-IFITM3KO cells were exposed to IFN-β (10 ng/ml, 16 h). The fractions of early endosomes (EE) and late endosomes/lysosomes (LE) were enriched from postnuclear supernatants (PNS) through sucrose gradient separation and analyzed for their cholesterol levels. Data are expressed as mean cholesterol concentrations (in micrograms per milliliter) ± SEM from at least three independent gradients. **, *P* ≤ 0.01; ns, not significant (two-way ANOVA with Tukey’s multiple-comparison test).

To assess the role of LE/L cholesterol as a host cell factor, we first determined whether IFN-β induces changes in LE/L cholesterol pools. Therefore, we used filipin, a highly fluorescent polyene that specifically binds free cholesterol and is well established as a histochemical marker for the detection of unesterified cellular cholesterol. Filipin stained the plasma membrane as well as intracellular compartments in A549 wild-type control cells (A549-WT), whereas cholesterol was prominently detected in vesicular structures in the perinuclear region in IFN-β-treated cells (10 ng/ml, 16 h). This is especially evident in the heat map representation, where the pixels (px) are encoded by different colors due to their intensities (Fig. 1A). To verify that the stronger perinuclear filipin signal was due to elevated levels of LE/L cholesterol rather than to a general expansion of this compartment, we next determined the degree of overlap of the LysoTracker Red DND-99 signals with the filipin signals using Manders’ coefficient. This method identifies those pixels which are doubly positive for both signals and calculates the ratio of their summed signal intensities from one channel to the total intensity in this channel. Thus, a greater coefficient is a good indicator of greater numbers of doubly positive pixels. Because the analysis was performed on z-stacks acquired from confocal microscopy, an axial dimension to each pixel was added, thus including the entire volume of the cell. As shown in Fig. 1A, this quantitative colocalization analysis confirmed that a significantly higher number of LysoTracker-positive pixels were also positive for filipin in IFN-treated cells than in control cells. Similar results were obtained when the colocalization analysis was performed using CD63 as a marker for LE/L (see Fig. S1A in the supplemental material), underscoring the idea that cholesterol pools are indeed increased in LE/L upon IFN exposure. Additionally, we have related the number of those voxels that are positive for both filipin and LysoTracker or CD63 signals to the total number of LysoTracker-positive (or CD63-positive) voxels. For both LE/L markers, the ratio is increased after IFN treatment, indicating a higher number of filipin-positive voxels which are also positive for the LE/L markers, regardless of the intensity of the filipin or Lysotracker/CD63 signal strength (Fig. S1B).

10.1128/mBio.01345-18.1 FIG S1  IFN-β induces endosomal/lysosomal cholesterol accumulation. (A) A549-WT cells left untreated or exposed to IFN-β (10 ng/ml, 16 h) were stained for LE/L marker protein CD63. Unesterified cellular cholesterol was visualized using filipin (blue). Filipin pixel intensities were pseudocolored according to the LUT and are shown as a heat map, with the gradient of colors ranging from blue (lowest intensity) to white (highest intensity). Bar, 20 µm. Colocalization coefficients of CD63 with filipin were quantitated from z-stacks. Mean values ± SEM were calculated from 35 individual cells per condition from at least two independent experiments. ****, *P* ≤ 0.0001 (unpaired Student’s *t* test). (B) Numbers of voxels positive for both filipin and LysoTracker or for filipin or CD63 individually were related to the total number of LysoTracker-positive (or CD63-positive) voxels. Mean values ± SEM were calculated from 35 individual cells per condition from at least two independent experiments. **, *P* ≤ 0.01 (unpaired Student’s *t* test). Download FIG S1, PDF file, 2.2 MB. Copyright © 2018 Kühnl et al. 2018 Kühnl et al. This content is distributed under the terms of the Creative Commons Attribution 4.0 International license.

Overexpression of the IFN-inducible antiviral protein IFITM3 has been linked to LE/L cholesterol accumulation (6, 10), and yet the functional relevance of this link, especially in the context of IFN-driven viral restriction, is not fully understood (6, 9–12). To functionally dissect the impact of LE/L cholesterol and IFITM3, we assessed whether the IFN-driven increase in LE/L cholesterol pools was still present in A549 IFITM3 knockout cells (A549-IFITM3KO), generated through the CRISPR/Cas9 system. Notably, these cells were able to respond to IFN, as assessed by IFN-mediated STAT1 induction, and yet were completely devoid of IFITM3 even under IFN stimulation conditions (Fig. S2A). In contrast, the homologous IFITM2 protein, which displays 91% sequence similarity (15) and also colocalizes with lysosomes (8, 16), was still upregulated in IFN-stimulated A549-IFITM3KO cells and yet the level of upregulation was very moderate (Fig. S2B). Although IFITM2 was also reported to induce cholesterol accumulation in endosomes (10), very weak IFN-stimulated IFITM2 induction, which was previously observed in the A549 cell line (17), did not compensate for the loss of IFITM3, as the IFITM3KO cells did not accumulate LE/L cholesterol upon IFN treatment (Fig. 1B). Consistently, colocalization of filipin with the LysoTracker signals was significantly decreased in the IFITM3KO cells after IFN treatment (Fig. 1B). To further substantiate these observations biochemically, we quantified and compared cholesterol levels in cell fractions enriched for either early endosomes or LE/L through sucrose gradient flotation separation of untreated or IFN-exposed cells. As shown in Fig. 1C, in both IFN-treated A549-WT and IFITM3KO cells, the cholesterol amounts in the postnuclear supernatants and in the early endosomal fractions were not significantly altered upon IFN treatment. However, IFN-treated A549-WT cells had significantly more cholesterol in the LE/L-enriched fractions than were seen with the untreated cells, whereas in IFITM3KO cells, no such increase was observed in this fraction, in line with the results obtained from the quantitative colocalization analysis. Together, these results show that IFN induces retention of cholesterol within LE/L and underscore the importance of IFN-driven upregulation of IFITM3 in this process, thereby confirming previous reports (10).

10.1128/mBio.01345-18.2 FIG S2  A549 IFITM3 knockout by CRISPR/Cas9 editing. (A) IFITM3 protein levels in lysates of A549-WT and A549-IFITM3KO cells either left untreated or exposed to IFN-β (10 ng/ml, 16 h) were analyzed by immunoblotting. Equal levels of protein loading were verified using GAPDH antibody. STAT1 served as a positive control for IFN-induced gene expression. (B) An antibody recognizing both IFITM2 and IFITM3 was used to analyze IFITM2 protein levels in the A549-IFITM3KO cells. GAPDH served as a loading control. (C) A549-IFITM3KO cells exposed to U18666A (U18) (2 µg/ml, 16 h) were stained for cellular cholesterol using filipin and were analyzed by confocal microscopy. Filipin pixel intensities were pseudocolored according to the LUT and are shown as a heat map, with the gradient of colors ranging from blue (lowest intensity) to white (highest intensity). Bar, 20 µm. Download FIG S2, PDF file, 1.2 MB. Copyright © 2018 Kühnl et al. 2018 Kühnl et al. This content is distributed under the terms of the Creative Commons Attribution 4.0 International license.

**FIG 2:**
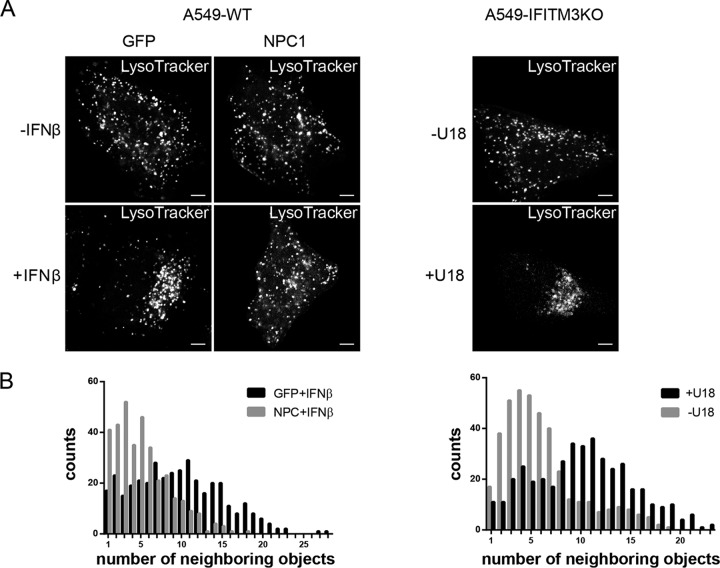
LE/L positioning is linked to LE/L cholesterol. (A) A549-WT cells transfected with GFP or YFP-NPC1 were left untreated or exposed to IFN-β (10 ng/ml, 16 h). A549-IFITM3KO cells were left untreated or exposed to U18666A (U18) (2 µg/ml, 16 h). All cells were then loaded with LysoTracker Red DND-99 to label acidic endosomes. Bar, 5 µm. (B) From each LysoTracker-positive object, the number of neighbors in a 30-px radius was determined with the help of the ImageJ plugin collection “BioVoxxel Toolbox.” Data are expressed as the frequencies of distribution determined for at least 5 cells per condition.

Interestingly, we observed that LysoTracker-positive compartments clustered in IFN-treated A549-WT cells, whereas they failed to do so in IFN-treated IFITM3KO cells. However, the IFITM3KO cells did not display a general defect in endosomal repositioning, as the clustered filipin-positive endosomes were seen upon treatment with 2 µg/ml of the hydrophobic polyamine U18666A, a cell-permeable small-molecule inhibitor of the endosomal cholesterol transporter NPC1 (18, 19), which is widely used as a pharmacological model to induce the NPC1 loss-of-function phenotype of LE/L cholesterol accumulation (Fig. S2C). Because this observation indicated that the endosomal clustering seen upon IFN treatment was dependent on the IFITM3-mediated increase in the LE/L cholesterol load, we next overexpressed NPC1. As shown in Fig. 2A, NPC1 overexpression in resting A549-WT cells did not alter the intracellular distribution of acidic endosomes. However, the IFN-induced LE/L clustering was clearly affected; IFN-treated A549-WT cells transfected with NPC1 displayed a LE/L distribution that resembled that in untreated cells, while the IFN-induced clustering was clearly recognizable in the green fluorescent protein (GFP)-transfected control cells. To check whether the LE/L of IFITM3KO cells, which do not show IFN-induced clustering, can be repositioned by forced LE/L cholesterol loading, we treated IFITM3KO cells with U18666A. In line with the notion that IFN-induced LE/L cholesterol accumulation was the reason for LE/L clustering, these cells showed similar redistributions of LysoTracker-positive endosomes after U18666A treatment (Fig. 2A). To further substantiate the importance of endosomal cholesterol levels in the LE/L positioning, we studied the distribution of the acidic endosomes more systematically. Therefore, we used software-assisted image analysis to quantify the number of neighbors (within a 30-px radius) of each LysoTracker-positive endosome in the individual cells. As shown in Fig. 2B, the endosomes of the IFN-treated A549-WT cells tended to have a greater number of neighbors, i.e., they were more densely crowded than acid endosomes in the untreated cells. Similarly, in the U18666A-treated IFITM3KO cells, the number of neighbors per acidic endosome was increasing. In summary, these results clearly show the dependency of LE/L positioning on LE/L cholesterol content.

### Interferon-independent LE/L cholesterol accumulation inhibits early IAV infection.

**FIG 3:**
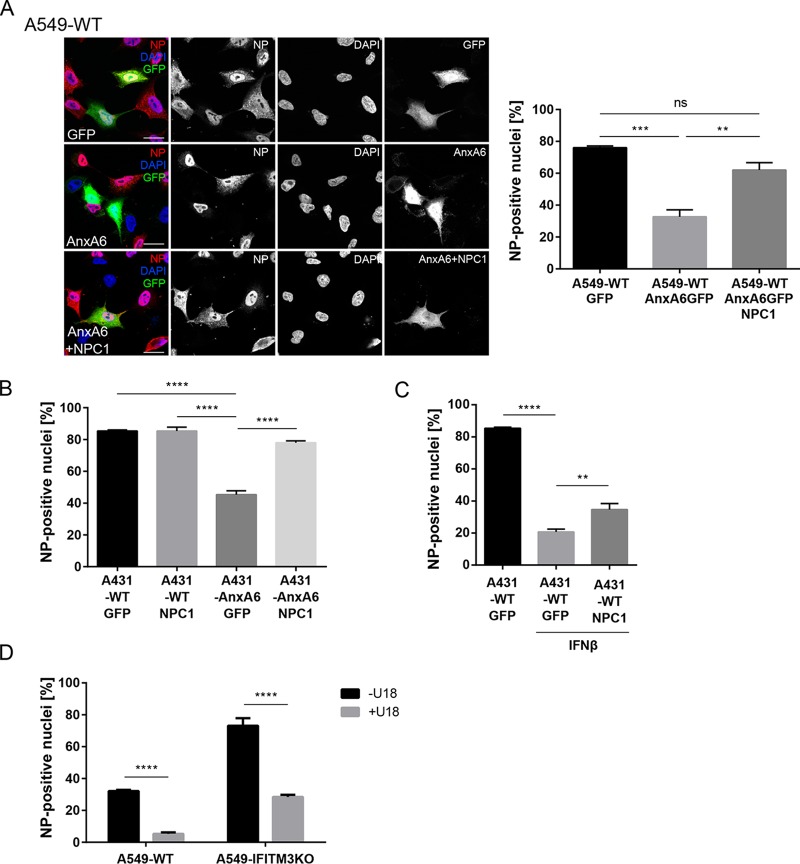
Interferon-independent LE/L cholesterol accumulation impairs early IAV infection. (A) A549-WT cells transfected with GFP, AnxA6-GFP, or AnxA6-GFP together with YFP-NPC1 were infected with IAV (PR8M; MOI of 5, 4 h). Cells were stained with anti-NP antibodies, and nuclei were labeled with DAPI. Bar, 20 µm. Numbers of transfected cells with NP-positive nuclei were determined from 150 transfected cells per condition from at least three independent experiments. Data are expressed as mean percentages ± SEM. **, *P* ≤ 0.01; ***, *P* ≤ 0.001; ns, not significant (one-way ANOVA with Tukey’s multiple-comparison test). (B) A431-WT and A431-AnxA6 cells were transfected with GFP or YFP-NPC1 as indicated and were infected with IAV (PR8M; MOI of 20, 4 h). Numbers of transfected cells with NP-positive nuclei were determined from 150 transfected cells per condition from three independent experiments. Data are expressed as mean percentages ± SEM. ****, *P* ≤ 0.0001 (two-way ANOVA with Tukey’s multiple-comparison test). (C) A431-WT cells expressing GFP or YFP-NPC1 were stimulated with IFN-β (10 ng/ml, 16 h) as indicated and then infected with IAV (MOI of 20, 4 h). Numbers of cells with NP-positive nuclei were determined from 150 transfected cells per condition from at least three independent experiments, and the mean percentages ± SEM of NP-positive nuclei were quantitated. *, *P* ≤ 0.05 (unpaired Student’s *t* test). (D) A549-WT and A549-IFITM3KO cells were exposed to U18666A (U18) (2 µg/ml, 16 h) and were infected with IAV (PR8M; MOI of 2, 4 h), stained for NP, and analyzed by FACS (10.000 cells per sample). Data are expressed as the mean percentages ± SEM of NP-positive nuclei from three independent experiments. ****, *P* ≤ 0.0001 (two-way ANOVA with Tukey’s multiple-comparison test).

To assess whether the IFN-induced increase in LE/L cholesterol levels was a side effect or a vital part of the IFN antiviral protection, we investigated LE/L cholesterol imbalance independently of IFN. For analysis of its physiological functions during the early stages of the infection, we aimed at impairing the egress of cholesterol from LE/L. To focus on early infection, we determined the proportion of infected cells within the first replication cycle. Therefore, nuclei of infected cells were analyzed 4 h postinfection (p.i.) for the appearance of the viral nucleoprotein (NP), which is predominantly nuclear at this stage of the infection cycle. Because NPC1 is a key element in the cholesterol transport from this compartment to other cellular destinations, increased cholesterol levels were first induced via overexpression of AnxA6, a negative regulator of NPC1 (13,20–22). As expected, a high proportion (76.0% ± 1.15%) of the GFP-positive control cells was infected, as revealed by the nuclear appearance of NP. In contrast to that, a much lower number of cells ectopically overexpressing AnxA6-GFP were NP positive (32.67% ± 4.37%). In line with the notion that enhanced LE/L cholesterol negatively affects IAV early infection, the proportion of infected cells strongly increased (62.0% ± 4.62%) when NPC1 was transiently coexpressed together with AnxA6 in these cells (Fig. 3A). To validate this finding, we next employed an established cell model consisting of the parental A431-WT cell line naturally lacking endogenous AnxA6 and of the A431-AnxA6 cell line engineered to stably express AnxA6 and thus showing an excess of LE/L cholesterol (13, 20–22). Both cell lines were infected with PR8 and monitored for NP staining 4 h p.i. As shown in Fig. 3B, the number of cells with NP-positive nuclei was decreased by approximately 50% in A431-AnxA6 cells compared to A431-WT cells. Strikingly, ectopic NPC1 expression in WT cells did not change the percentage of infected cells, whereas, in the A431-AnxA6 cells, NPC1 expression almost completely rescued the reduced proportion of infected cells in the single cycle (Fig. 3B). This suggests that an optimal concentration of cholesterol in LE/L membranes is required for efficient early infection. Next, we compared the numbers of NP-positive cells in IFN-β-primed A431-WT cells ectopically expressing the tagged cholesterol transporter NPC1 or empty vector (GFP). As expected, infection of nontreated A431-WT cells expressing GFP yielded a high number of infected cells, whereas IFN-β pretreatment drastically reduced the numbers of NP-positive nuclei (Fig. 3C). Of note, NPC1 overexpression significantly increased the amounts of NP-positive nuclei in IFN-β-primed A431-WT cells. Thus, NPC1 overexpression counteracted the host-protective role of IFN, supporting the idea that the IFN/IFITM3-induced increase in LE/L cholesterol is a part of the innate immune response against IAV.

In the second experimental approach, we treated A549-WT cells with the NPC1 inhibitor U18666A. To exclude the possibility that any U18666A effect was due to an induction of IFITM3-mediated cholesterol imbalance, the A549-IFITM3KO cells were investigated in parallel. Because the proportion of infected cells was increased in these cells, a lower virus titer was used in order to maintain a dynamic assay window also in the A549-IFITM3KO cells. A drastically reduced number of NP-positive nuclei were detected in U18666A-treated A549-WT cells (Fig. 3D). Importantly, U18666A treatment had the same negative effect on early IAV infection in the IFITM3KO cells (which are able to accumulate cholesterol in LE/L under conditions of treatment with U18666A; see Fig. S2C), indicating that LE/L cholesterol accumulation exerts a host-protective function independently of IFITM3 induction. As shown in Fig. S3A, the U18666A concentration that was used to induce LE/L cholesterol accumulation (2 µg/ml) did not affect the endosomal low pH, a key factor for IAV cell entry. Similarly, overexpression of IFITM3 (Fig. S3B) or AnxA6 (Fig. S3C) had no effect on endosomal acidification, indicating that the observed viral restriction in situations characterized by elevated LE/L cholesterol content is not caused by impaired endosomal acidification.

10.1128/mBio.01345-18.3 FIG S3  Characterization of endosomal pH. Endosomal/lysosomal pH was measured by ratio imaging of OG488/TMR-dextran in A549-WT cells incubated overnight with the indicated concentrations of U18666A (U18) (A) as well as in A549-WT cells transfected with myc-IFITM3 or empty vector (B) and in A431-WT and A431-AnxA6 cells (C). pH values are means ± SEM of results from 30 cells from five independent experiments. ****, *P* ≤ 0.0001; ns, not significant (one-way ANOVA followed by Tukey’s multiple-comparison test). Download FIG S3, PDF file, 0.04 MB. Copyright © 2018 Kühnl et al. 2018 Kühnl et al. This content is distributed under the terms of the Creative Commons Attribution 4.0 International license.

In summary, in NPC1-inhibited A549 cells, through either AnxA6 expression or U18666A treatment, the cholesterol imbalance in the LE/L compartment restricted the first infection cycle of incoming IAV particles, independently of the antiviral properties of IFITM3.

### LE/L cholesterol accumulation inhibits IAV/endosome membrane fusion.

**FIG 4:**
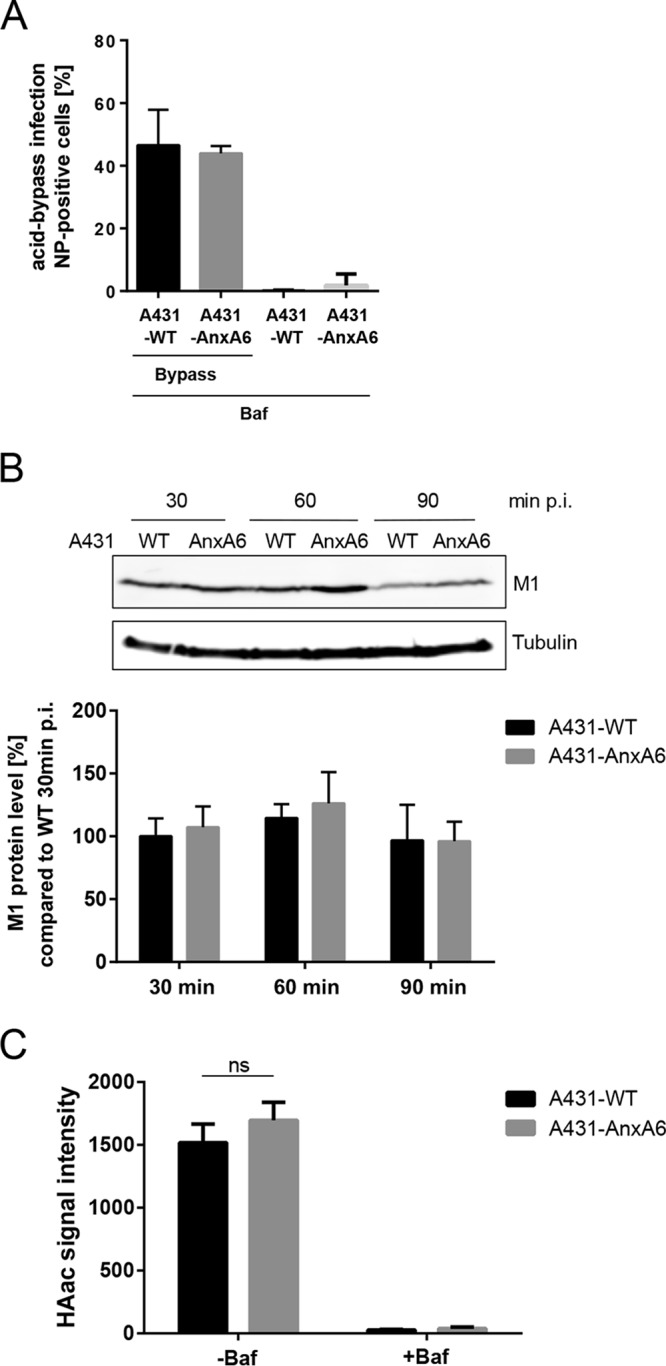
Impact of LE/L cholesterol accumulation on the sequential steps of IAV host cell entry. (A) A431-WT and A431-AnxA6 cells were infected with IAV (PR8M; MOI of 50) at 4°C for 1 h. An acidic bypass was applied to induce fusion at the plasma membrane, and infection through endosomal uptake was blocked by bafilomycin A1 (Baf) treatment. Cells were further incubated for 8 h, stained for NP, and analyzed by FACS for NP-positive cells. Data are expressed as the mean percentages ± SEM of NP-positive cells from three independent experiments. (B) A431-WT and A431-AnxA6 cells were infected with IAV (PR8M; MOI of 10). M1 protein levels were monitored by Western blotting, and blots were probed for tubulin to verify equal levels of loading. Mean M1 signal intensities ± SEM of results from three independent experiments were calculated relative to the mean M1 intensity detected in A431-WT cells 30 min p.i. (C) A431-WT and A431-AnxA6 cells were infected with IAV (×31; MOI of 20). Cells were fixed and stained 1 h p.i. with the A1 antibody to detect the acid-induced conformation of HA (HAac). The signal intensity per cell was quantified by ImageJ analysis. Mean values ± SEM were calculated from 55 individual cells per condition from at least two independent experiments. ns, not significant (two-way ANOVA followed by Tukey’s multiple-comparison test).

To unravel the details of the actual step that was negatively affected by increased LE/L cholesterol levels, we first performed acid-bypass infection assays. This technique forces the fusion of IAV particles with the host cell plasma membrane (23) and leads to a direct transfer of the viral genome into the cytosol, thus circumventing the endocytic pathway. Strikingly, there was no difference in the numbers of infected A431-WT and A431-AnxA6 cells (Fig. 4A), suggesting that LE/L cholesterol accumulation acted on the endosomal uptake of IAV. We next performed a comprehensive analysis of the sequential events during host-cell entry. In line with our previous work (13), the levels of efficiency of IAV endosomal uptake, as judged by the levels of the virion-associated matrix protein M1 (24), were similar in A431-WT and A431-AnxA6 cells (Fig. 4B). Because the acidic environment encountered in the LE/L induced conformational changes in HA that critically affect the fusogenic capacity, we next used an antibody specific for the fusion-active conformation (25). As expected from the findings indicating that LE/L pH was not affected, no significant differences in the amount of signal were detected in A431-WT and A431-AnxA6 cells (Fig. 4C). In contrast, hardly any signal could be detected in cells treated with bafilomycin A1, an inhibitor of the vacuolar ATPase required for endosomal acidification, which therefore served as a positive control.

**FIG 5:**
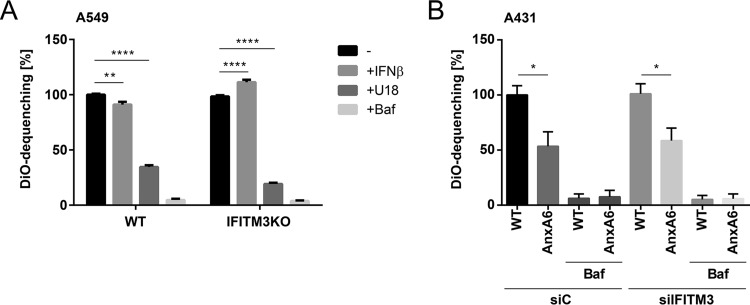
LE/L cholesterol accumulation inhibits IAV/host membrane fusion. (A) A549-WT and A549-IFITM3KO cells were either left untreated or exposed to IFN-β (10 ng/ml, 16 h), U18666A (U18) (2 µg/ml, 16 h), or bafilomycin A1 (Baf) and were infected with dually labeled IAV (PR8M; MOI of 10). SP-DiOC-positive cells were identified by FACS analysis at 1 h p.i. (10,000 cells per condition). Data are expressed as percentages of the mean number of SP-DiOC-positive cells in the untreated A549-WT cell sample and represent mean values ± SEM. **, *P* ≤ 0.01; ****, *P* ≤ 0.0001 (two-way ANOVA followed by Tukey’s multiple-comparison test). (B) A431-WT and A431-AnxA6 cells transfected with control (siC) or IFITM3-specific (siIFITM3) siRNA were either left untreated or incubated with bafilomycin A1 (Baf) and were infected with dually labeled IAV (PR8M; MOI of 10). SP-DiOC-positive cells were identified by FACS analysis at 1 h p.i. (10,000 cells per condition). Data are expressed as percentages of the mean number of SP-DiOC-positive cells in the untreated A431-WT cell sample and represent mean values ± SEM. *, *P* ≤ 0.05 (two-way ANOVA followed by Tukey’s multiple-comparison test).

Transfer of the viral genome into the cytosol occurs during passage through the late endosomal compartment. Hence we speculated that LE/L cholesterol imbalance might interfere with viral uncoating and/or membrane fusion. Therefore, a viral envelope/endosomal membrane fusion assay utilizing IAV particles labeled with the two lipophilic dyes 3,3′-dioctadecyl-5,5′-di(4-sulfophenyl)oxacarbocyanine (SP-DiOC18) and octadecyl rhodamine b chloride (R18), initially described by Sakai et al. (26), was employed to identify potential fusion defects. Upon fusion, the dyes diffuse out of the viral envelope and into the endosomal membrane, leading to dequenching and a subsequent increase in SP-DiOC18 fluorescence. This elevated SP-DiOC18 signal was decreased in infected A549-WT cells transiently overexpressing IFITM3 compared to nontransfected control cells, verifying that the assay was suited for detection of antiviral restriction through host cell factors (Fig. S4A). Bafilomycin A1, which blocks endosomal acidification, an essential prerequisite for successful IAV membrane fusion, almost completely abolished SP-DiOC18 dequenching in both A549-WT and A549-IFITM3KO cells (Fig. 5A). Remarkably, the IFN treatment resulted in a decrease in dequenching in the A549-WT cells, whereas in the A549-IFITM3KO cells the opposite effect, i.e., an increase in dequenching, was found. Importantly, in both cell lines, U18666A treatment significantly caused a pronounced decrease in SP-DiOC18 dequenching, indicating that IAV membrane fusion within cholesterol-laden endosomes was impaired. Similar results were observed when A431-WT cells and A431-AnxA6 cells were compared for IAV-endosome fusion, and the impaired virus fusion with cholesterol-laden endosomal membranes was still observed in A431-AnxA6 cells that were subjected to small interfering RNA (siRNA) treatment to silence IFITM3 expression (Fig. 5B). Because these results contradicted previously published findings on the impact of LE/L cholesterol on viral envelope/endosomal membrane fusion (6), we applied the respective single-dye dequenching approaches used in the study by Desai et al. Notably, in this assay, neither use of SP-DiOC18 nor use of 1,1′-dioctadecyl-3,3,3′,3′-tetramethylindodicarbocyanine,4-chlorobenzenesulfonate (DiD) yielded significant differences in the efficiencies of IAV/endosomal membrane fusion between A431-AnxA6 cells and A431-WT cells, although robust bafilomycin A1-mediated inhibition could be detected (Fig. S4B). This suggests that single-dye fusion assays display reduced sensitivities.

10.1128/mBio.01345-18.4 FIG S4  Characterization of impaired IAV endosomal escape. (A) A549-WT cells transfected with myc-IFITM3 or empty vector as a control were infected with IAV particles labeled with SP-DiOC and R18 (PR8M; MOI of 10). To monitor lipid mixing of virus envelope and endosomal membranes, dequenching of SP-DiOC was measured by FACS analysis at 1 h p.i. (10,000 cells per condition). Data are expressed as percentages of the mean numbers of SP-DiOC-positive cells in the empty vector control and represent mean values ± SEM of results from three independent experiments. *, *P* ≤ 0.05 (Student’s *t* test). (B) A431-WT and A431-AnxA6 cells were either left untreated or incubated with bafilomycin A1 (Baf) and were infected with single-labeled IAV (PR8M; MOI of 10). SP-DiOC-positive cells or DiD-positive cells were identified by FACS analysis at 1 h p.i. (10,000 cells per condition). Data are expressed as percentages of the mean number of SP-DiOC-positive cells or DiD-positive cells in the untreated A431-WT cell sample and represent mean values ± SEM. (C) A549-WT cells were either left untreated or exposed to U18666A (U18) (2 µg/ml, 16 h) and/or bafilomycin A1 (Baf) and were infected with dually labeled IAV (PR8M; MOI of 10). SP-DiOC-positive cells were identified by FACS analysis at the indicated times p.i. (10,000 cells per condition). Data were expressed as geomeans of SP-DiOC signals in the respective samples, and the slopes of linear regression were calculated. Histograms of the original FACS results are depicted at the bottom of the panel. Histogram marker M1 defines the SP-DiOC-positive cell subpopulation. Download FIG S4, PDF file, 0.4 MB. Copyright © 2018 Kühnl et al. 2018 Kühnl et al. This content is distributed under the terms of the Creative Commons Attribution 4.0 International license.

In order to investigate the influence of the pharmacologically increased LE/L cholesterol level in more detail, we followed the progress of the fusion over time (Fig. 4C). U18666A-treated cells showed lower values at all times measured during the 6 h after infection. However, the analysis of the dequenching rates revealed that the increases in signal intensity were approximately parallel in both cases. This most likely reflects the well-known fact that the bulk of the fusion occurs within the first hour after infection (25) and that the propagation of the dye in the host membrane after fusion is not disturbed by U18666A.

Thus, our results point to an inhibitory effect of LE/L cholesterol on viral envelope-endosomal membrane fusion, further supporting our findings indicating that LE/L cholesterol accumulation is an efficient restriction factor operating at the step of endosomal escape and independently of IFITM3.

## DISCUSSION

A solid body of evidence supports the idea of the importance of cholesterol in host cell membranes as a factor that influences propagation of many viruses (10, 27–29), and our previous work uncovered imbalanced cellular cholesterol homeostasis as an important factor for IAV particle production at the plasma membrane and infectivity of released viral particles (13). Now, we report that the accumulation of cholesterol in LE/L interferes with IAV infection at a much earlier stage, i.e., at the step of endosomal escape. In line with previous reports, we show that antiviral IFN leads to IFITM3 upregulation and concomitant IFITM3-dependent LE/L cholesterol accumulation. Importantly, elevating LE/L cholesterol pools independently of IFN and IFITM3 via AnxA6 overexpression or pharmacological inhibition of NPC1 had the same antiviral consequences, even when IFITM3 expression was abolished through targeted genome editing.

The induction of the interferon system is a universal element in the activation of the host cell innate immune response against viruses. When cells sense viral components, interferons produced in infected cells are secreted to induce an antiviral state in surrounding cells, thus keeping viral spread at bay. Among the host cell proteins induced by interferon, IFITM3 is a potent antiviral factor that strongly restricts IAV (5–12, 30). The antiviral activity of IFITM3 is mechanistically not fully resolved. IFITM3 is detected at LE/L (8) and was reported previously to induce the accumulation of LE/L cholesterol (10, 11), possibly through interfering with ER-LE/L contact sites (10). Although the impact of IFITM3 on membrane fusion has been observed by several studies (6, 8, 9), the role of LE/L cholesterol accumulation as part of antiviral IFITM3 activity has been questioned (6, 11). LE/L cholesterol accumulation has been observed in IFITM3-overexpressing cells (10), but the issues of whether IFN treatment itself would also result in upregulation of LE/L cholesterol contents (via upregulation of IFITM3 or, rather, independently of IFITM3) and whether such IFN-driven increased LE/L cholesterol levels would restrict IAV endosomal escape had not been addressed previously (12). Here, we report that IFN treatment indeed elevates the amounts of LE/L cholesterol. Interestingly, such an increase of the levels of LE/L cholesterol upon IFN treatment was suggested earlier but was not shown experimentally (10). The IFN-driven LE/L cholesterol increase appeared to depend on the upregulation of IFITM3, in line with previous data obtained from cells ectopically overexpressing IFITM3.

We also noted a striking repositioning of LE/L after IFN treatment which was dependent on IFITM3 and increased LE/L cholesterol content and which could be mimicked in IFITM3KO cells through forced cholesterol loading of LE/L. The results clearly demonstrated a dependency of LE/L positioning on LE/L cholesterol content.

Because the antiviral impact of LE/L cholesterol overload on IAV cell entry is heavily disputed (6, 9–12), we utilized AnxA6 overexpression and the small-molecule inhibitor U18666A to interfere with the proper function of the endosomal cholesterol transporter NPC1. Both approaches represent alternative IFN-independent and well-established models used to induce LE/L cholesterol accumulation (reviewed in reference 14). We identified that the interferon-independent LE/L cholesterol accumulation was clearly antiviral and restricted early IAV infection in cells not exposed to IFN-β, thus supporting the idea that LE/L cholesterol is an element of the IFN response chain. NPC1 was discovered as an entry factor for filoviruses (31, 32) and in the case of the Ebola virus entry apparently acts in a cholesterol-independent fashion (31). Our findings indicating that NPC1 overexpression *per se* did not increase the proportion of IFN-β-unprimed, IAV-infected cells argue, however, against a cholesterol-independent function for NPC1 in IAV entry. The significantly impaired number of NP-positive nuclei seen during infection of AnxA6-overexpressing A431 cells as well as A549 cells indicated that early IAV infection was affected. An earlier analysis by Ma et al. did not detect decreased NP staining using a similar cell system. However, it is not known whether the AnxA6-mediated LE/L cholesterol phenotype was apparent in their stable cell line (33). IFITM3-sensitive viruses, such as IAV, are still able to reach the acidic endosomes, the site where pH-dependent viral envelope/membrane fusion occurs (8, 34). In agreement, we did not observe inhibition of IAV uptake (as judged by the presence of IAV M1 protein) and trafficking in U18666A-treated or AnxA6-overexpressing cells.

Like most enveloped viruses (3), IAV exploits the drop in pH encountered in the LE/L as a trigger for viral envelope-endosomal membrane fusions (4), in turn leading to the cytosolic release of the viral genome. Low pH induces insertion of the HA fusion peptide into the endosomal membrane, pulling the two membranes together (3, 4). The two bilayers merge partially (hemifusion) and eventually fuse completely, thereby creating and expanding a fusion pore. For IAV, low pH is sufficient to trigger this fusion process. Although impaired endosomal acidification has been linked to LE/L cholesterol imbalance (6), we did not observe significant alterations in the LE/L pH and in the amount of HA that underwent the fusion-active conformational change in cells with impaired NPC1 functionality, indicating that changes in the lipid composition of the target membrane and not compromised endosomal acidification most likely caused the impaired fusion of the viral envelope with the endosomal membrane. LE/L are typically pleiomorphic and contain intraluminal vesicles within their limiting membrane (35). Endosomal cholesterol overload has been reported to impair intraendosomal membrane trafficking, i.e., the back-fusion of intraluminal vesicles with the limiting membrane (27). Accordingly, infection with vesicular stomatitis virus (VSV), a virus that first fuses with the intraluminal vesicles to then release the viral genome via back-fusion of the nucleocapsid-containing internal vesicles (36), is impaired when cells are forced to accumulate late endosomal cholesterol (27). We observed a strikingly negative impact of increased LE/L cholesterol levels on virus/endosome lipid mixing. Thus (hemi)fusion between the viral envelope and endosomal membranes was impaired. Notably, acidic bypass-induced IAV fusion at the plasma membrane, which circumvents passage through LE/L compartments (23), was not affected, underscoring the fact that defective endosomal escape is the cause of IAV entry inhibition in AnxA6-expressing cells displaying elevated LE/L cholesterol levels. Interestingly, inhibition of IAV HA-mediated hemifusion (9) was reported upon IFITM3 overexpression, although it was attributed to a higher lipid order of the host membranes, and a potential involvement of IFITM3-induced elevation of cholesterol content was not investigated. However, since a cell-cell fusion assay using HA-expressing cells was used in this study, there is a possibility that the actual HA-mediated viral envelope/endosomal membrane fusion was not fully reproduced.

HA-mediated membrane fusion has been addressed in many studies employing biophysical approaches, and special attention has been paid to the role of cholesterol in this process (see reference 37 for an overview). Membrane fusion is dynamic and is influenced by the lipid composition of both the host cell target membrane and the viral envelope (38), and *in vitro* liposome studies reflect neither the membrane curvature nor the exact lipid (and protein) composition in endosomes. Here, we focused on the impact of increased LE/L cholesterol levels observed only under certain physiological conditions, i.e., during the IFN-mediated antiviral response. Free cholesterol within LE/L is thought to diffuse preferentially into membranes of the intraluminal vesicles (39), and endocytosed IAV particles might also incorporate the excess cholesterol into the viral envelope, which might in turn disturb proper HA mobility and/or function. In support of this idea, a moderate level of removal of cholesterol from the IAV envelope has been shown to enhance fusion (38). The IAV fusion peptide is an α-helical hairpin structure (3), and changes in the lipid composition of the target membranes might interfere with the proper insertion. It is also highly likely that LE/L cholesterol enrichment affects the curvature and/or rigidity of the endosomal membranes, causing restricted fusion capacity.

The data presented in this study contradict previous studies that reported LE/L cholesterol accumulation and reduced IAV endosomal escape in IFITM3-overexpressing cells but did not detect an effect on virus-membrane hemifusion, leading to the assumption that excess LE/L cholesterol is not the mechanism by which IFITM3 inhibits the IAV fusion. Similarly, only a minor impact on lipid mixing was observed when the LE/L compartment was subjected to cholesterol loading using U18666A (6). However, in the study mentioned, pseudovirus infection was used. More importantly, our comparison of the different methods used to measure lipid mixing (single dye versus dual dyes) indicated that single-dye fusion assays might have reduced sensitivity. Consistently, we always detected a pronounced decrease in hemifusion in U18666A-treated cells, applying the well-established dual-dye assay (23, 25, 26, 30). Thus, our results point to an inhibitory effect of LE/L cholesterol on viral envelope/endosomal membrane fusion. Besides its activity with respect to LE/L cholesterol contents, IFITM3 was proposed to affect IAV endosomal escape at a step later than hemifusion, possibly by negatively affecting fusion pore formation and opening (6, 11). We did not observe impaired membrane fusion in cells that had not been treated with interferon but that were downregulated for IFITM3, and yet the number of infected cells was increased. These observations suggest that IFITM3 has several roles in the complex process of IAV endosomal escape, in addition to cholesterol-mediated inhibition of hemifusion. In turn, this could explain the fact that no rescue due to overexpression of NPC1 in IFITM3 overexpressing cells was seen in earlier studies (10).

Most of the IFN-induced antiviral effectors control viral replication, i.e., act at a stage when the virus genome is already found in the cytoplasm. Importantly, the cholesterol axis identified here efficiently interferes with the cytosolic entry of incoming viral particles, a very early step in the infection cycle. Impaired transfer of the viral genome into the cytoplasm might provide the host cell with more time to mount the antiviral response.

Taken together, our data establish the LE/L cholesterol balance as a functionally relevant effector that acts as an early antiviral barrier. The LE/L cholesterol-mediated restriction is embedded in the antiviral response mounted upon IFN-β exposure of host cells, with IFITM3 functioning as the IFN-controlled switch. A precise modulation of cholesterol at the step of incoming IAV might combine reduced primary infection and fewer side effects and therefore could open up novel antiviral approaches targeting host factor components, and LE/L cholesterol levels might serve as a likely host target.

## MATERIALS AND METHODS

### Biological materials.

The A431 human epithelial carcinoma cell line stably expressing AnxA6 (A431-AnxA6; provided by T. Grewal, Sydney) has been described previously (40). The A431 wild-type cells (A431-WT; provided by T. Grewal, Sydney), the A431-AnxA6 cells, and the A549 human alveolar epithelial cell line (provided by S. Ludwig, Muenster) were cultivated in Dulbecco’s modified Eagle’s medium (DMEM). Madin-Darby canine kidney (MDCKII) cells (provided by S. Ludwig, Muenster) were cultured in minimal essential medium (MEM). Cell culture media were supplemented with 10% fetal bovine serum (Merck catalog no. S0615), 100 U/ml penicillin, and 0.1 mg/ml streptomycin. Cells were cultured at 37°C in a 5% CO_2_ atmosphere. For IFN pretreatment, cells were exposed to 10 ng/ml IFN-β (recombinant human interferon beta; ImmunoTools catalog no. 11343524) for 16 h.

### Transient transfection of plasmids and siRNA.

A549 and A431 cell lines were transfected with plasmid using Lipofectamine 2000 (Invitrogen) according to the manufacturer’s protocol. pCMV-myc-hIFITM3 was kindly provided by Jacob Yount (Addgene plasmid number 58461). Human AnxA6 was expressed from the plasmid pEGFP-N1 (41), and murine NPC1 was expressed from the plasmid pEYFP-N2 (42). pEGFP-N3 served as a control.

### CRISPR/Cas9-mediated genome editing.

IFITM3 was knocked out in A549 cells through the use of gene editing plasmids from Santa Cruz. The IFITM3-specific CRISPR/Cas9 plasmid was cotransfected with the IFTM3-specific HDR plasmid, and KO cells were positively selected in puromycin-containing (2 µg/ml) selection medium. Basal IFITM3 expression and IFN-induced IFITM3 expression in WT and IFTM3KO cells were compared by Western blotting using an anti-IFITM3 antibody (Proteintech catalog no. 11714-1-AP). To verify that IFN signaling was not affected in the IFITM3 KO cells, IFN-mediated STAT1 induction (43) was monitored by Western blotting (Cell Signaling catalog no. 9172). Detection of GAPDH (glyceraldehyde-3-phosphate dehydrogenase) (Santa Cruz catalog no. sc-25778) served as a control to confirm equal levels of protein loading. siRNA against human IFITM3 (SMART pool siGENOME, human IFITM3 siRNA M-014116-01; GE Dharmacon) was used for knockdown of IFITM3 protein expression in A431-WT and A431-AnxA6 cells, and nontargeting siRNA (ON-Target plus siControl; GE Dharmacon) served as a negative control.

### IAV infection.

H1N1 IAV strain A/Puerto Rico/8/34 (PR8M) was obtained from the virus strain collection of the Institute of Virology, University of Muenster, Muenster, Germany, and H3N2 IAV strain X31 was provided by Thorsten Wolff, Robert Koch Institut, Berlin, Germany. Both IAV strains were propagated in MDCKII cells. For infection, cells were washed with phosphate-buffered saline (PBS) and exposed to the indicated multiplicities of infection (MOIs) of virus diluted in PBS with 0.2% bovine serum albumin (BSA; Sigma catalog no. A7979), 1 mM MgCl_2_, 0.9 mM CaCl_2_, 100 U/ml penicillin, and 0.1 mg/ml streptomycin (PBS-BSA) at 37°C or on ice. If not otherwise indicated, the inoculum was aspirated after 30 min. Cells were then washed with PBS and incubated in DMEM with 0.2% BSA, 100 U/ml penicillin, and 0.1 mg/ml streptomycin (DMEM-BA) for the respective periods of time.

### Subcellular staining and colocalization analysis.

Cells were grown on coverslips and fixed with 4% paraformaldehyde (PFA)–PBS^++^ (PBS with Ca^2+^/Mg^2+^) for 10 min at room temperature. For cholesterol staining, cells were further incubated with filipin (filipin complex from Streptomyces filipinensis; Sigma catalog no. F9765) (stock, 2.5 mg/ml in dimethyl sulfoxide [DMSO] and 1:2 in 2% BSA) for 2 h. For visualization of endosomal/lysosomal compartments, cells were incubated either with 200 nM LysoTracker Red DND-99 (Molecular Probes) for 1 h prior to fixation or with an anti-CD63 antibody (MX-49.129.5; Santa Cruz catalog no. sc-5275) (tetramethylrhodamine [TMR] isocyanate [TRITC]; 1:100 in 2% BSA) for 1 h after filipin staining. Confocal microscopy was performed using an LSM 780 microscope (Carl Zeiss, Inc., Jena, Germany) equipped with a Plan-Apochromat 63×/1.4 oil immersion objective. z-stacks of individual cells were subjected to threshold processing, and Manders’ colocalization coefficients of the respective endosomal/lysosomal marker with filipin signal were analyzed using the ImageJ plugin Jacop (44). Additionally, the numbers of voxels that were positive for both filipin and LysoTracker (or for filipin and CD63) were related to the total number of LysoTracker-positive (or CD63-positive) voxels, regardless of the intensity of the filipin or LysoTracker/CD63 signal strength. To generate the heat map presentations of filipin stainings, the different filipin pixel intensities were color-coded according to the gradient presented in a color look-up table (LUT), with the gradient of colors ranging from blue (lowest intensity) to white (highest intensity).

To analyze the clustering of acidic endosomes, the grayscale images were processed in a binary manner by replacing LysoTracker intensity values above the globally determined threshold with a value of 1 and setting all other values to 0. With the help of the ImageJ plugin collection “BioVoxxel Toolbox,” the resulting binary images were then used to determine the number of neighbors of each endosome in the individual cells, and the data were expressed as frequency distributions of at least 5 cells per condition.

### Cell lysis and Western blotting.

For protein sample preparation, cells were washed in PBS, harvested in 8 M urea, and subjected to sonication. The protein concentration in the lysates was determined by the Bradford method. The primary antibodies used for detection of the respective proteins by SDS-PAGE and Western blotting were anti-annexin VI antibody (N-19; Santa Cruz catalog no. sc-1931), anti-GAPDH antibody (FL-335; Santa Cruz catalog no. sc-25778), anti-IFITM3 antibody (Proteintech; 11714-1-AP), anti-IFITM2/3 antibody (Proteintech; 66081-1-Ig), anti-influenza M1 antibody (clone GA2B; AbD Serotec [MCA401]), and anti-α-tubulin antibody (clone B-5-1-2; Sigma catalog no. T5168). IRDye secondary antibodies (Li-COR) labeled with near-infrared (NIR) fluorescent dyes were used for direct, nonenzymatic detection of primary antibodies coupled with IRDye 680CW and IRDye 800CW for donkey anti-mouse IgG (H+L), donkey anti-rabbit IgG (H+L), and donkey anti-goat IgG (H+L). An Odyssey infrared imaging system (Li-COR) was used for NIR fluorescence detection. Western blots were quantified using Odyssey infrared imaging system software version 3.0.25.

### Single-cycle infection analysis.

Cells were pretreated as indicated in the respective figure legends, seeded on covers slips 1 day prior to infection, and infected with PR8M by a standard procedure as described above. At 4 h p.i., cells were fixed (4% PFA–PBS^++^ for 10 min), permeabilized (0.1% Triton X-100–PBS^++^ for 30 min), and blocked (2% BSA–PBS^++^, overnight, 4°C). Staining with anti-NP antibody (mouse anti-influenza A virus nucleoprotein, clone AA5H; AbD Serotec [MCA400]) for 2 h and anti-mouse Alexa Fluor 594 (donkey anti-mouse IgG [H+L] secondary antibody, Alexa Fluor 594; Invitrogen catalog no. A21203) for 1 h with DAPI (4′,6-diamidino-2-phenylindole) (Sigma) was done using 2% BSA. Transfected cells were identified by confocal microscopy using an LSM 800 microscope (Carl Zeiss, Inc., Jena, Germany) equipped with a Plan-Apochromat 63×/1.4 oil immersion objective and were categorized for the presence or absence of distinct IAV NP signal.

### Acidic bypass.

The applied assay was established previously by Stauffer et al. (23). Prior to infection, cells were preincubated with bafilomycin A1 (Cayman catalog no. cay-11038) (250 nM). Virus (PR8M) was primed for 1 h at 37°C with DMEM adjusted to pH 5.8 with MES (morpholineethanesulfonic acid) (30 mM). Cells were infected at an MOI of 50 with primed virus for 1 h on ice and subsequently washed twice with cold DMEM-BA. An acidic bypass was achieved by incubation with low-pH DMEM (50 mM citrate, pH 5.0) for 2 min, followed by two washing steps with cold DMEM-BA. DMEM-BA containing bafilomycin A1 (250 nM) was added, and cells were incubated for 8 h at 37°C. For fluorescence-activated cell sorter (FACS) analysis, the cells were harvested by Accutase treatment (Millipore catalog no. SCR005), fixed in 4% formaldehyde for 10 min, and permeabilized by the use of 0.1% Triton X-100 for 30 min. Blocking as well as staining with an anti-NP antibody (mouse anti-influenza A virus nucleoprotein, clone AA5H; AbD Serotec [MCA400]) for 2 h and anti-mouse Alexa Fluor 488 (donkey anti-mouse IgG secondary antibody) (Alexa Fluor 488; Invitrogen catalog no. R37114) for 1 h was done using 2% BSA–PBS^++^. Cells were washed three times between blocking and antibody incubations. A total of 10,000 cells were analyzed per sample. Uninfected cells or cells without bypass served as a control.

### Detection of virus-associated M1 protein.

Early detection of virus-associated M1 protein to monitor IAV internalization was performed as previously described (24). Briefly, cells were infected with PR8M at an MOI of 5 for 30 min at 37°C as described above. After the standard infection procedure, cells were washed with acidic PBS (PBS/HCl; pH 1.3, 4°C) to remove noninternalized virions from outside the cell. Subsequently, cells were washed twice with PBS (4°C) and incubated with DMEM-BA at 37°C. At the indicated time, cells were lysed and analyzed by Western blotting against IAV M1 protein and α-tubulin as a loading control.

### Acid-induced conformational change of HA.

The detection of HA after the acid-induced conformational change was performed as described previously (25, 45). An antibody which specifically recognizes HA that has undergone an acid-induced conformational change (HAac) was kindly provided by Yohei Yamauchi (ETH Zürich).

### Subcellular fractionation and cholesterol quantification.

LE/L membrane fractions were enriched via sucrose density gradient centrifugation as described previously (46, 47), and cholesterol was quantified via the use of an Amplex Red cholesterol assay kit (Invitrogen) as described earlier (13).

### Endosomal pH measurement.

Ratiometric fluorescence microscopy and calculation of endosomal pH were done as described by Johnson et al. (48). Briefly, cells were pulsed for 25 min with Oregon green 488 (OG488)-labeled and tetramethylrhodamine (TMR)-labeled dextran (Invitrogen) (10 kDa), followed by a 25-min chase. Cells were washed and kept in HEPES-buffered Hanks' balanced salt solution (HBSS) at 37°C during image acquisition. Epifluorescence signal was acquired for each of the dyes individually and at intervals of 60 s. To generate a calibration curve based on the mean OG488/TMR fluorescence ratio at the respective pH, standard solutions ranging from pH 4.5 to 6.0 were applied after basal measurement, and endosomal pH was calculated from the calibration curve.

### Virus fusion assay.

Virus lipid labeling using 3,3′-dioctadecyl-5,5′-di(4-sulfophenyl)oxacarbocyanine (SP-DiOC18; Molecular Probes catalog no. D7778) and octadecyl rhodamine b chloride (R18; Molecular Probes catalog no. O246) was described previously (25, 26) and was adapted to the single use of SP-DiOC18 or 1,1′-dioctadecyl-3,3,3′,3′-tetramethylindodicarbocyanine,4-chlorobenzenesulfonate (DiD; Molecular Probes catalog no. D7757). In brief, IAV (PR8M) was labeled with SP-DiOC18 and R18 at final concentrations of 0.2 µM and 0.4 µM, respectively, or with SP-DiOC18 or DiD alone at 0.2 µM for 1 h at room temperature and was subsequently filtered through a 0.22-µm-pore-size filter (Millipore). Cells were infected with the labeled virus for 30 min on ice, washed twice with PBS, and subsequently incubated for the indicated periods of time at 37°C. Cells were harvested by Accutase (Millipore catalog no. SCR005) treatment and were fixed with 2% formaldehyde for 20 min. Lipid mixing was quantified by FACS analysis, with 10,000 cells analyzed per sample. Linear regression analysis (GraphPad Prism) was used to determine the best-fit linear regression and to calculate the slopes.

### Statistical analysis.

*A priori* power analysis (G*Power 3.1) (49) was used to estimate the sample size required to detect moderate to strong effects. Data are given as means ± standard deviations (SEM). Statistical significance of the results was evaluated by unpaired Student’s *t* test or analysis of variance (ANOVA [one-way or two-way]) followed by Tukey’s multiple-comparison test using GraphPad Prism version 4.00 (GraphPad Software, Inc., San Diego, CA).
