# Supplementary figures and images for: Late Endosomal/Lysosomal Cholesterol Accumulation Is a Host Cell-Protective Mechanism Inhibiting Endosomal Escape of Influenza A Virus
Source: mBio. 2018 Jul 24;9(4):e01345-18. doi: 10.1128/mBio.01345-18 (PMC6058292; doi:10.1128/mBio.01345-18)

A

A549-WT

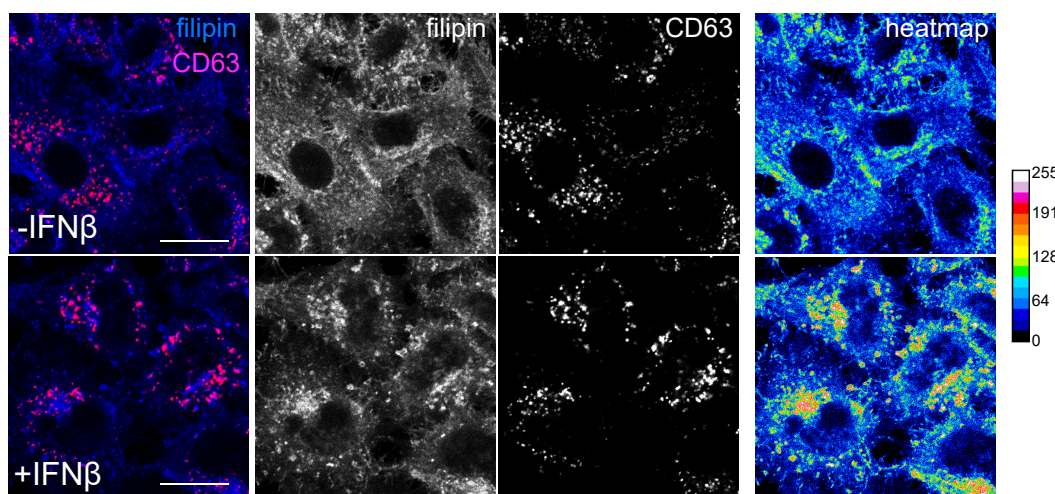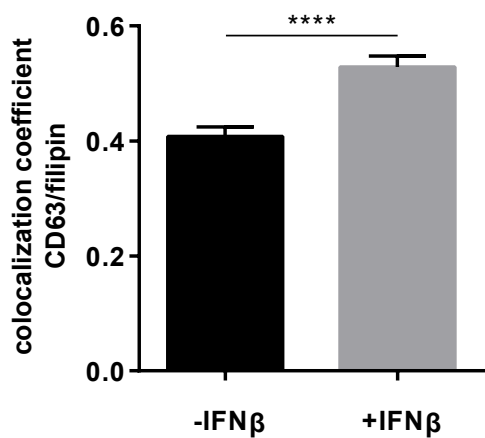

B

A549-WT

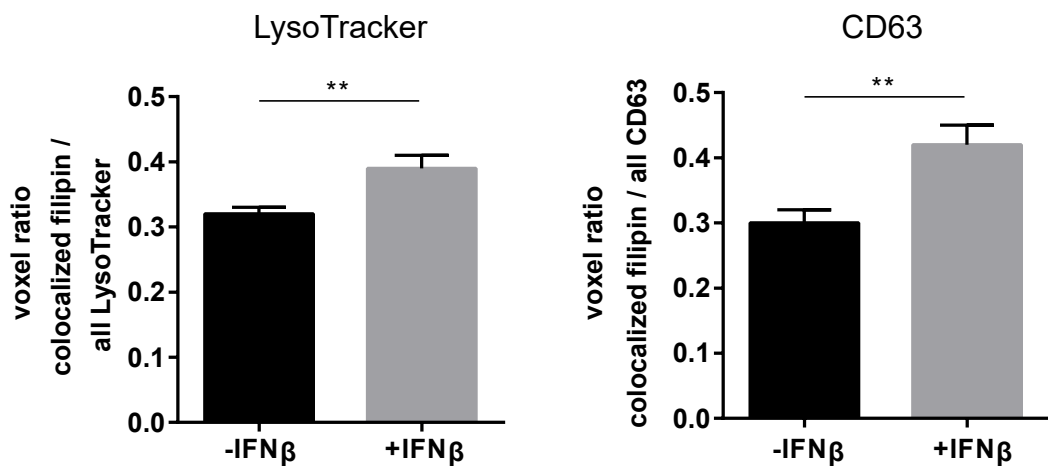

Supplement: FIG S1 [file mbo004183990sf1.pdf]

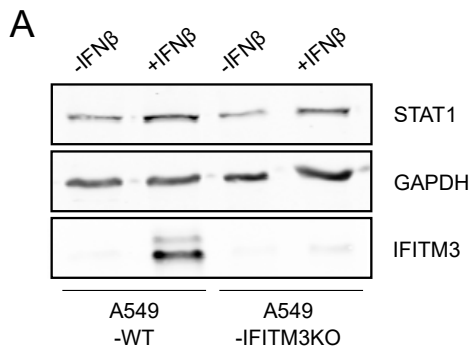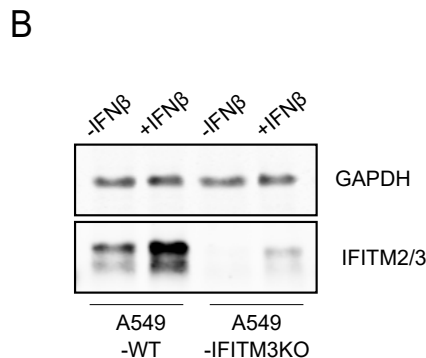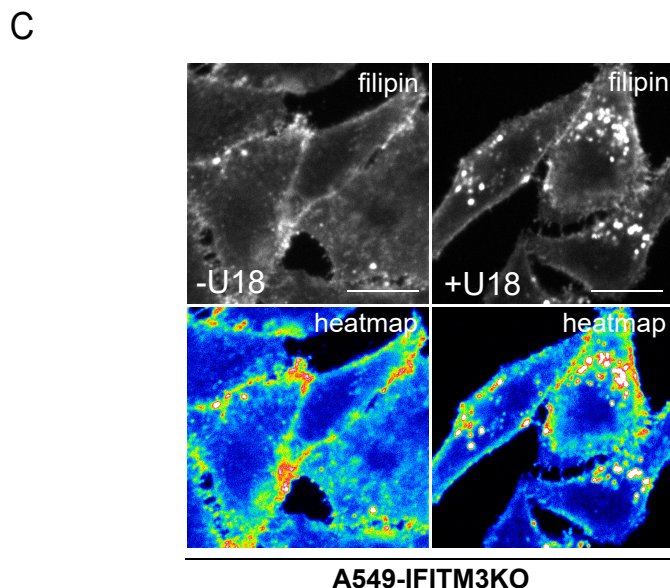

Supplement: FIG S2 [file mbo004183990sf2.pdf]

# Kuehnl et al., Figure S3

A

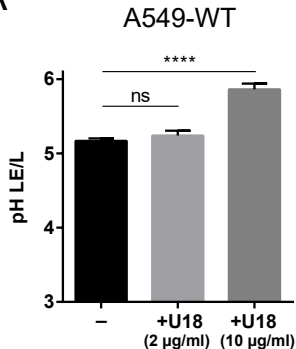

B

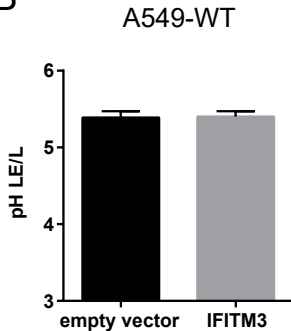

C

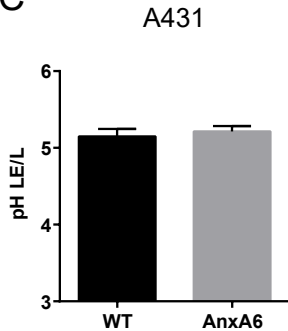

Supplement: FIG S3 [file mbo004183990sf3.pdf]

A

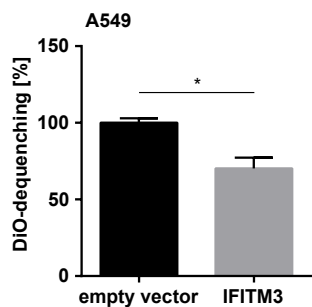

B

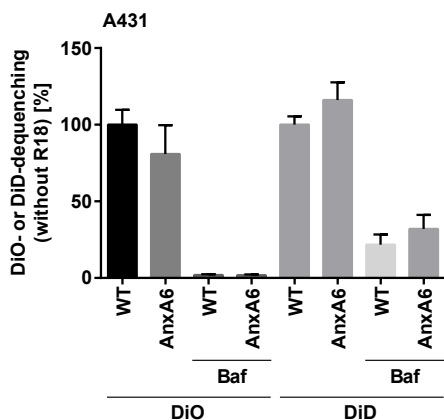

C

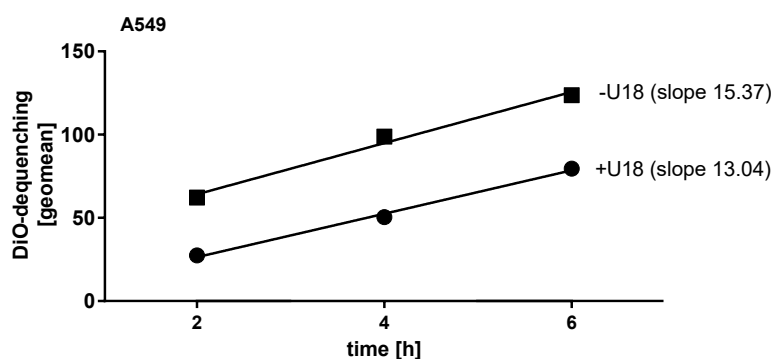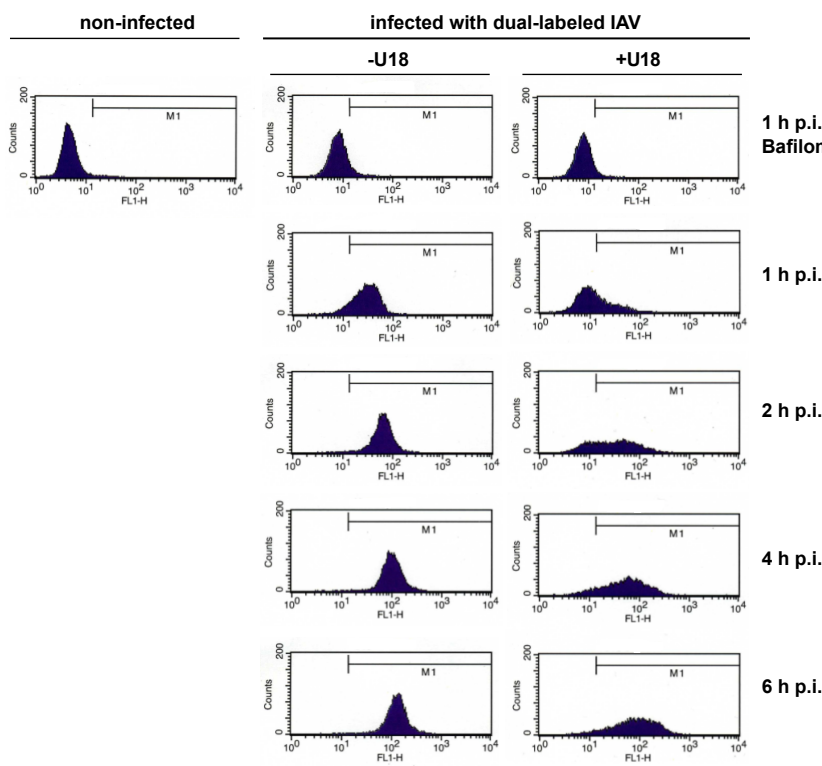

Supplement: FIG S4 [file mbo004183990sf4.pdf]
